# Pan-Plastome Analysis Reveals the Genetic Diversity and Genetic Divergence of *Adenocaulon himalaicum* (Asteraceae)

**DOI:** 10.3390/ijms26178594

**Published:** 2025-09-04

**Authors:** Nan Lin, Yuxuan He, Xiankun Wang, Yakun Wang, Jinhao Wang, Yang Li

**Affiliations:** 1 College of Life Science, Henan Agricultural University, Zhengzhou 450046, China; yuxuanhhee@outlook.com (Y.H.); w987361763@163.com (Y.W.); wjh3295957935@163.com (J.W.); 2 Henan Engineering Research Center for Osmanthus Germplasm Innovation and Resource Utilization, Henan Agricultural University, Zhengzhou 450046, China; 3 State Key Laboratory of Plant Diversity and Specialty Crops, Kunming Institute of Botany, Chinese Academy of Sciences, Kunming 650201, China; 4 College of Landscape Architecture and Art, Henan Agricultural University, Zhengzhou 450046, China; wangxiankunx@163.com

**Keywords:** *Adenocaulon himalaicum*, comparative genomics, genetic differentiation, genetic structure, pan-plastome

## Abstract

The pan-plastome approach provides a powerful tool for investigating intraspecific divergence and population genetics due to its unique advantages, including single-copy genes, absence of recombination, and moderate nucleotide substitution rates. *Adenocaulon himalaicum* Edgew. (Asteraceae), a widely distributed medicinal herb in East Asia, remains genomically understudied at the population level, with no comprehensive pan-plastome assembly available to date. Here, we de novo assembled pan-plastome of 87 individuals from 18 populations representing its known distribution range. The pan-plastome exhibited a typical quadripartite structure (152,129 bp to 152,207 bp), containing 113 unique genes, most of which were under purifying selection. Phylogenetic and haplotypes analyses revealed three distinct genetic lineages corresponding to their geographic distribution. Population genetic analyses showed significant differentiation among three genetic groups (AMOVA: 73.43% variation among groups) and a strong isolation-by-distance pattern (IBD: r = 0.469, *p* = 0.001). The pronounced population structure underscores the imperative for establishing distinct conservation units, with particular emphasis on marginal populations that may harbor unique genetic compositions and potential medicinal properties. Our study presents the first pan-plastome for *A. himalaicum*, offering new insights into its plastome evolution and population divergence, providing valuable genomic resources to guide future breeding and sustainable utilization of medicinal herbs.

## 1. Introduction

The genus *Adenocaulon* Hook. (Asteraceae) comprises about five species with a disjunct distribution in Asia and America [1]. Members of this genus exhibit unique evolutionary traits within Asteraceae, particularly its distinctive seed morphology lacking a pappus, which represents an excellent system for studying intraspecific divergence in angiosperm [2]. *Adenocaulon himalaicum* Edgew. is the only species within this genus exhibiting an exceptionally broad distribution across East Asia [1]. This species demonstrates remarkable ecological plasticity, thriving in diverse shaded, mesic environments across an extensive elevational gradient from lowlands to subalpine zones [3]. Morphologically, *A. himalaicum* is characterized by erect stems (ca. 30–100 cm) bearing arachnoid hairs and stipitate glands on upper portions. The leaves of the adaxial surface appear subglabrous while the abaxial surface is densely covered with whitish arachnoid hairs [3]. Notably, the winged petioles and progressively reduced upper leaves that transition into broadly lanceolate bract-like structures represent key diagnostic features. These morphological adaptations, combined with its extended reproductive period and glandular structures, collectively enhance its capacity to colonize diverse habitats.

In addition to its ecological significance, *A. himalaicum* has been widely utilized in traditional Chinese medicine owing to its diverse array of bioactive compounds [4]. Pharmacological studies have identified its major classes of therapeutic metabolites, such as acetylenic glycosides, monoterpene glycosides, and caffeic acid derivatives [5,6,7]. These compounds contribute to the remarkable medicinal properties, particularly its detoxification capacity and anti-inflammatory effects, which have been empirically utilized in folk medicine for generations [8]. Despite the wide geographical distribution across East Asia, *A. himalaicum* faces increasing threats to its genetic resources. Rapid habitat fragmentation caused by agricultural expansion, urbanization, and other human activities has led to progressive decline of natural populations [9,10]. The habitat loss raises concerns about genetic erosion, particularly given the knowledge gaps that persist regarding the genetic diversity pattern of the populations [11,12].

Despite the growing interest in medicinal plant genomics and the extensive plastome sequencing efforts within Asteraceae [13,14,15], the plastome evolution of *A. himalaicum* remains understudied. This not only hinders our understanding of the species’ evolutionary history and population genetics, but also limits potential biotechnological applications of its valuable medicinal compounds [5]. The plastome has emerged as a powerful tool for population genetics studies in medical plants due to its conserved genomic architecture, uniparental inheritance pattern, and minimal recombination events [16,17]. Compared to traditional studies relying on limited genetic fragments (e.g., *ITS*, *matK*, or *trnL-F*), plastomes provide substantial phylogenetic resolution and more accurate estimates of genomic diversity by leveraging hundreds of variable loci [18,19,20]. While prior studies on *Adenocaulon* and related Asteraceae were constrained by low-information markers (less than five genetic fragments), the plastome approach can uncover deep lineage splits that were previously undetectable [14]. Empirically, plastome analyses of *Dolomiaea* DC. and *Robinsonia* DC. resolved distinct phylogenetic lineages and identified population-specific adaptive mutations, demonstrating how plastome data can transform previously unresolved taxonomic groups into well-defined conservation units [21,22].

Recently, the pan-plastome concept has emerged as a more comprehensive and precise approach for investigating genetic variation at the intraspecific level [23]. In contrast to plastomes constructed from single or a few representative individuals, the pan-plastome integrates genomic data across multiple individuals from diverse origins, facilitating the distinction between conserved core genomes and polymorphic variable regions [13,24,25]. This approach provides a comprehensive framework for analyzing genetic diversity, cryptic lineage divergence, historical hybridization, or lineage admixture at the intraspecific level [23,25]. Therefore, the application of the pan-plastome in genetics studies is expected to yield crucial insights into the genetic diversity and population divergence of important medical plants.

To investigate the pan-plastome evolution and genetic pattern of *A. himalaicum* across East Asia, this study conducted comprehensive genetic analyses using the pan-plastome of *A. himalaicum*. We sequenced and assembled 87 complete plastomes from 18 populations across the distribution range, with three key objectives: (1) to characterize structural and evolutionary variations in *A. himalaicum* through pan-plastome analysis; (2) to assess intraspecific genetic diversity and genetic differentiation across the populations; and (3) to predict potential distribution shifts under paleoclimate change scenarios to provide supplementary protection suggestions. This research provides genomic resources for future studies of this medicinally valuable species and identifies patterns of genetic variation relevant for conservation and breeding.

## 2. Results

### 2.1. Comparative Analysis of the Pan-Plastome in A. himalaicum

**Figure 1:**
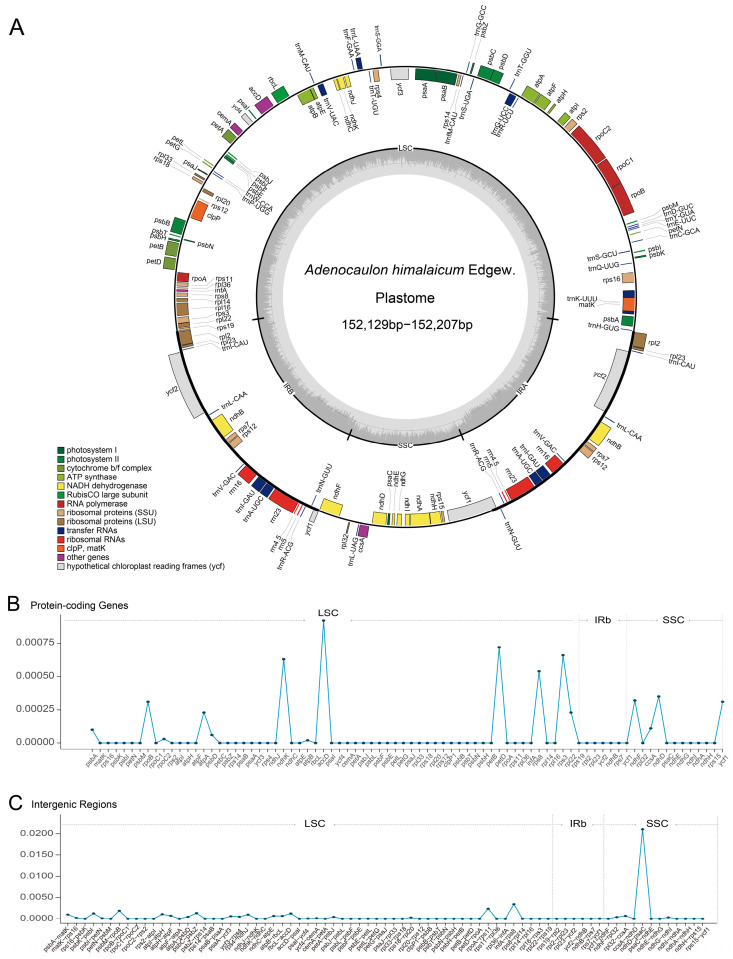
Pan-plastome map and comparison of nucleotide diversity (Pi) across all *A. himalaicum* individuals. (**A**) Pan-plastome map of *A. himalaicum.* Genes from different functional groups are shown in different colors in the outermost first ring. (**B**) Comparison of nucleotide diversity for coding regions and (**C**) non-coding regions of the pan-plastome.

The mean coverage depth of the 87 newly sequenced plastomes of *A. himalaicum* ranged from 14.5 to 4014.2 (Table S1). The pan-plastome from 87 individuals of *A. himalaicum* and one outgroup from *A. nepalense* exhibited a typical quadripartite structure (Figure 1A, Table S2), comprising a large single-copy (LSC) region, a small single-copy (SSC) region, and a pair of inverted repeats (IRA and IRB). The total genome size of *A. himalaicum* ranges from 152,129 bp to 152,207 bp, with an average length of 152,191 bp (Table S2). The LSC region ranges from 83,252 bp to 83,341 bp (average length: 83,320 bp), while the SSC and IR region ranges from 18,637 bp to 18,643 bp (average length: 18,641 bp) and 25,112 bp to 25,119 bp (average length: 25,115 bp). The overall GC content of the pan-plastome is 37.70%, with the IR regions having the highest GC content (43.16–43.17%). A total of 113 genes were annotated in the *A. himalaicum* plastomes, including 79 protein-coding genes, 30 tRNA genes, and 4 rRNA genes (Table S3). Among the protein-coding genes, three of those contain two introns (*clpP*, *rps12*, and *ycf3*). Additionally, a pseudogene (*accD*) was annotated in all individuals of *A. himalaicum*. Comparative analysis of the pan-plastome revealed that *ycf1* and *rpl2* genes were positioned at the quadripartite junctions, with *ycf1* in IRa-SSC (JSA) and *rpl2* in IRb-LSC (JSB). In addition, three inversion events were detected in the *A. himalaicum* pan-plastome: two previously documented inversions (a 22.8 kb inversion between *trnG-UCC* and *trnE-UUC*, and a 3.3 kb inversion between *trnE-UUC* and *trnC-GCA*), along with a novel 1.2 kb inversion spanning from *psbM* to *trnE-UUC* (Figure 1).

Nucleotide diversity (Pi) analysis across the 79 protein-coding genes revealed high sequence conservation (Figure 1B), with Pi values ranging from 0.00002 (*atpB*) to 0.00092 (*accD*). In contrast, non-coding regions exhibited a greater variation (Figure 1C), where Pi values spanned from 0.00002 (*psbE-petL*) to 0.02105 (*ndhD-psaC*). Based on PAML analysis, we demonstrated that all protein-coding genes had a nonsynonymous-to-synonymous substitution rate ratio (dN/dS) below 1, consistent with strong purifying selection (Figure S1).

### 2.2. Codon Usage Bias and Repeat Sequence Analysis

We analyzed codon usage frequency across all 79 protein-coding genes in the *A. himalaicum* pan-plastome (Figure S2), identifying a total of 22,805 codons per individual. These comprised 61 sense codons and 3 stop codons, encoding 20 amino acids. Arginine (Arg) and serine (Ser) were the most abundant amino acids (each representing 9.84% of the total), while tryptophan (Trp) was the least frequent (1.64%). Among the codons, 30 showed relative synonymous codon usage (RSCU) values >1, with UAA being the preferred stop codon.

Tandem repeat finder (TRF) analysis indicated repeat lengths ranging from 9 to 53 bp, with 9 bp repeats being the most common (Figure S3). Across the pan-plastome, we detected 2323 microsatellite (SSR) loci, predominantly located in intergenic regions, with only a minor fraction in coding regions (Table S4). SSR distribution was biased toward the LSC region, while the IR and SSC regions contained significantly fewer repeats. Mononucleotide repeats dominated (88.6%), followed by dinucleotide (7.6%) and trinucleotide repeats (3.7%). Notably, 7.4% of the SSRs were compound repeats (Figure S3). The most frequent mononucleotide and dinucleotide motifs were T and TA, respectively. REPuter analysis revealed that forward repeats were the most abundant, whereas reverse and complementary repeats were comparatively rare (Figure S3).

### 2.3. Population Structure and Haplotype Network Analysis

Based on the pan-plastome from 87 individuals, a total of 116 SNVs were identified, and all of these were biallelic sites (Table S5). These SNVs contained 22 singleton and 94 parsimony-informative sites. The majority of the SNVs were located in the LSC region (78, 67.20 %), followed by the SSC (28, 24.10 %), while the IR had the fewest variants (10, 8.60 %). There were 47 SNVs in spacer regions, 69 in the coding regions, and none in intron regions. The *ycf1* gene exhibited the highest number of SNVs (nine SNVs), followed by the *psaC* gene (five SNVs). In addition to SNV, we identified 36 indels in the *A. himalaicum* pan-plastome, including 8 microsatellite-related indels, 1 repeat-related indel, and 27 normal indels. Notably, all the indels were located in intergenic spacer regions.

**Figure 2:**
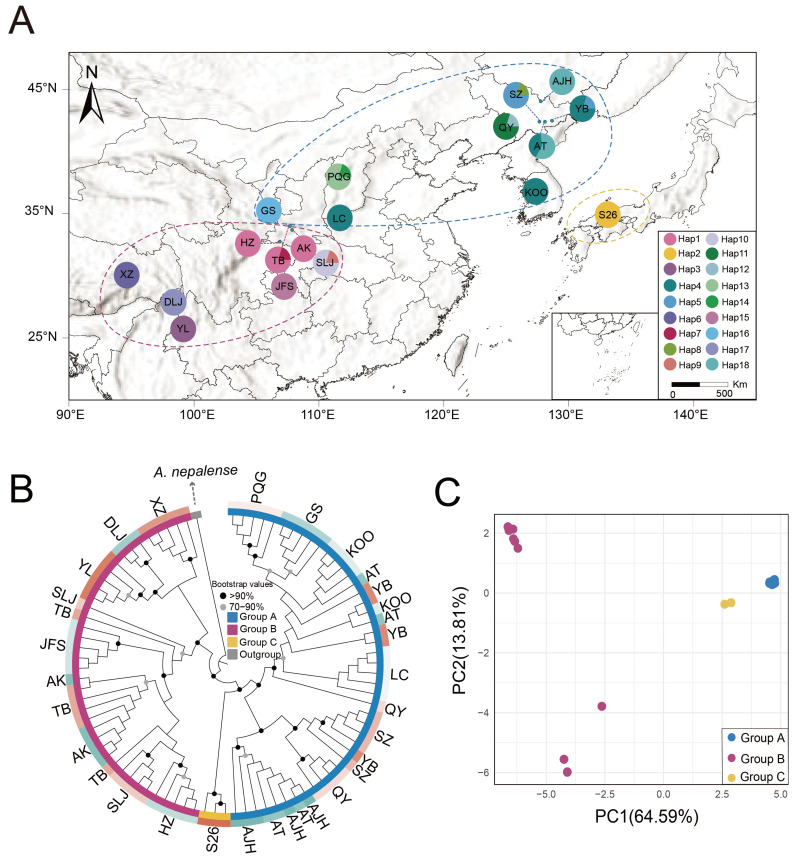
Population location, cpDNA haplotype distribution, and genetic clusters of *A. himalaicum* based on phylogeny and PCA. (**A**) Geographic distribution of cpDNA haplotypes, with color-coded populations corresponding to the haplotypes in the bottom right corner. (**B**) Maximum likelihood (ML) phylogenetic tree of *A. himalaicum* populations, with *A. nepalense* as outgroup. (**C**) Principal component analysis (PCA) showing the genetic clustering of *A. himalaicum* populations.

Phylogenetic analysis based on maximum likelihood (ML) and Bayesian inference (BI) using IQ-TREE v2.2 and MrBayes v3.2.7, resolved all *A. himalaicum* individuals into three well-supported genetic clades (Figure 2A and Figure S4). These clades correspond to distinct geographic regions: northeastern China and the Korean Peninsula (Group A), southern China (Group B), and Japan (Group C). While most populations formed monophyletic lineages, individuals from populations YB, AK, and TB exhibited a dispersed distribution pattern rather than clustering into distinct groups (Figure 2B). Principal component analysis (PCA) further supported the observed genetic structure, with the first two principal components (PC1 and PC2) explaining 64.59% and 13.81% of the total genetic variation, respectively (Figure 2C). PC1 clearly separated the southern clade (Group B) from the other populations, whereas PC2 differentiated the Japanese clade (Group C) from the northern clade (Group A). Additionally, populations XZ, YL, and DLJ from Group B were distinguished along the PC2 axis. Overall, the PCA results were consistent with the phylogenetic lineages, revealing pronounced genetic differentiation among populations.

**Figure 3:**
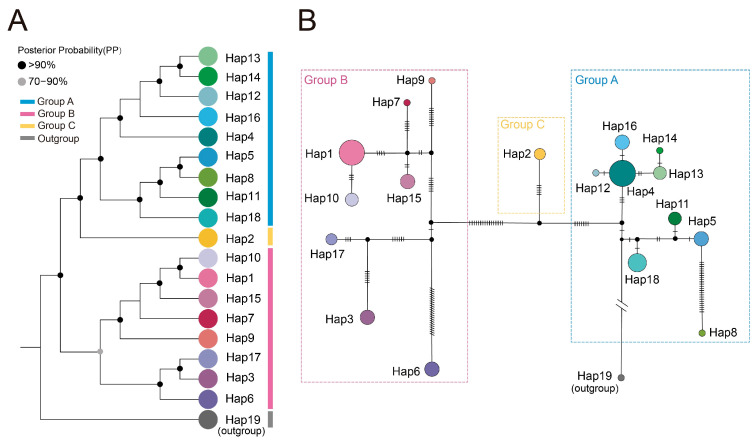
Phylogeny and haplotype network of 18 haplotypes in *A. himalaicum* with *A. nepalense* as outgroup. (**A**) Bayesian phylogenetic tree for cpDNA haplotypes for *A. himalaicum*. (**B**) The median-joining network of 18 cpDNA haplotypes in *A. himalaicum* with *A. nepalense* as outgroup (Hap19). The vertical short lines represent mutational steps. The sizes of circles are proportional to the number of haplotypes.

A total of 18 haplotypes were detected among the 87 *A. himalaicum* individuals, clustering into three distinct groups (Groups A, B, and C) that align with the phylogenetic and PCA results (Figure 3A and Figure S5). Most haplotypes were population-specific, with only four shared by multiple populations. Hap1 was shared by the population AK, TB, and HZ; Hap4 was found in the population AT, YB, LC, and KOO; and Hap5 was shared by population YB and SZ. According to the haplotype network (Figure 3B), 18 haplotypes formed three major clusters; this was consistent with the haplotype-based phylogeny, supporting the significant genetic differentiation patterns among *A. himalaicum* populations.

### 2.4. Analyses of Genetic Diversity and Genetic Differentiation

**Figure 4:**
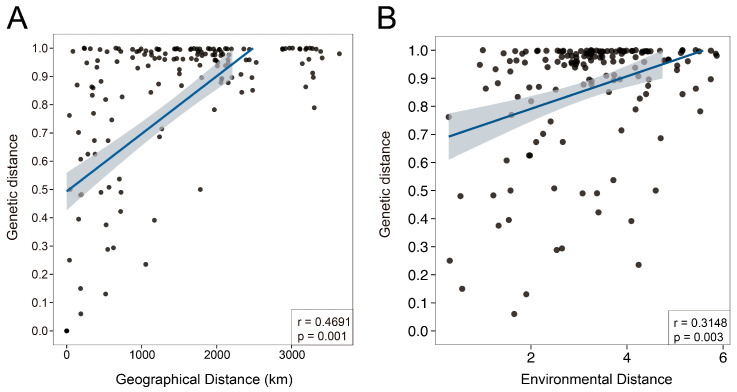
Analysis of Mantel tests between genetic distance and (**A**) geographical distance and (**B**) environmental distance based on linear regression. Shaded areas represent 95% confidence intervals of the regression lines.

Our analyses revealed a significant phylogeographic structure in *A. himalaicum*, with total *N*_ST_ (0.880) being significantly higher than *G*_ST_ (0.773; *p* < 0.05). Genetic diversity estimates showed modest nucleotide diversity (Pi = 0.0002) but high haplotype diversity (Hd = 0.913) across the 18 populations (Table S6). Analysis of molecular variance (AMOVA) demonstrated that predominant genetic variation (73.43%) occurred among the three major groups, with lower contributions from variations among populations (23.12%) and within populations (3.45%) (Table S7). Pairwise *F*_ST_ values indicated strong genetic differentiation between populations. Furthermore, Mantel tests showed significant positive correlations between genetic distance and both geographic distance (r = 0.469, *p* = 0.001) and environmental distance (r = 0.315, *p* = 0.003) (Figure 4).

### 2.5. Ecological Niche Modeling

**Figure 5:**
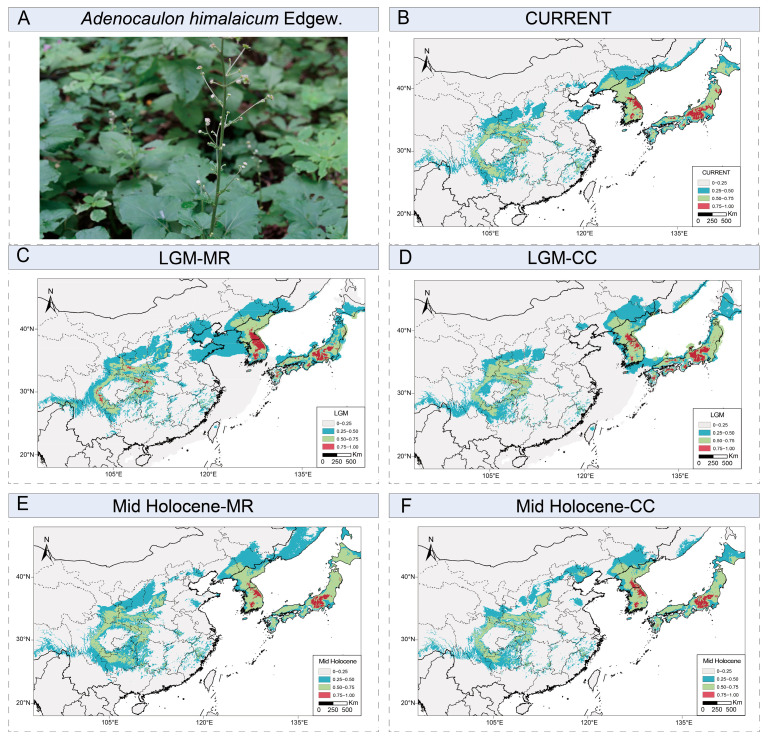
Ecological niche modeling of *A. himalaicum* under four different climate scenarios. (**A**) Morphology of *A. himalaicum* (**B**) current distribution (1970–2000), (**C**,**D**) Last Glacial Maximum (LGM, ca. 22,000 years ago), and (**E**,**F**) Mid-Holocene (ca. 6000 years ago) under two climate models (MIROC-ESM and CCSM4).

Ecological niche modeling under two paleoclimate scenarios (Last Glacial Maximum [LGM] and Mid-Holocene) exhibited robust predictive performance (AUC > 0.90), with generally consistent results across both of the two climate models (MIROC-ESM and CCSM4; Figure 5, Table S8). Predictions for the current distribution were generally good representations of the actual distributions of *A. himalaicum*, with core suitable habitats concentrated around the Sichuan Basin, the Korean Peninsula, and Japan (Figure 5B). Notably, during the LGM, highly suitable habitats in northern Japan shrank slightly compared to present conditions under CCSM4 and MIROC-ESM models (Figure 5C,D). However, the MIROC-ESM model suggested a minor expansion of moderately suitable habitats into northeastern China and the East China Sea land bridge. Projections during the Mid-Holocene indicated a reduction in highly suitable habitat areas compared to both present and LGM conditions.

## 3. Discussion

### 3.1. Pan-Plastome Characteristics and Evolution in the Adenocaulon himalaicum

In this study, analyses of the pan-plastome of 87 individuals of *Adenocaulon himalaicum* revealed highly conserved quadripartite structures, as reported in other species [26,27,28]. The genome size (152,129–152,207 bp), gene number (113 genes, 79 protein-coding genes, 30 tRNA, and 4 rRNA), and GC content (37.70%) align with previously reported plastome features in Asteraceae species, such as *Carthamus* L. [27], *Aster* L. [29], and *Artemisia* L. [15]. The expansion and contraction of inverted repeat (IR) regions represent a key mechanism driving plastome structural variation, influencing gene duplication, reduction, and pseudogenization [30,31,32]. Variations in IR regions have been reported in *Tetracentron* Oliv. [33] and *Pelargonium* L’Hér. ex Aiton [34], which found a larger number of gene expansions in the IRs. However, substantial gene losses in IRs were reported in Convolvulaceae Juss. [35], Taxaceae Gray. [36], Pinaceae Spreng. ex F. Rudolphi. [37], and Cupressaceae Gray. [38]. In contrast to these extreme cases, the plastome of *A.* *himalaicum* demonstrates only a single gene contraction in the IR region, characterized by the presence of *rpl2* at the IRa/LSC junction and *ycf1* at the IRb/SSC junction, respectively (Figure 1). This pattern is consistent with most Asteraceae species [14], underscoring the conserved feature of IR dynamics in this family.

Gene rearrangements are common in Asteraceae plastomes, occurring frequently across multiple lineages [39,40,41]. Notably, two inversions in the large single-copy (LSC) region, a 22.8 kb inversion between *trnG-UCC* and *trnE-UUC*, and a 3.3 kb inversion between *trnE-UUC* and *trnC-GCA*, have been widely documented in Asteraceae species [39]. In addition to these shared inversions, *A. himalaicum* displays a unique 1.2 kb inversion spanning from *psbM* to *trnE-UUC* in the LSC region. Although such rearrangements are rare in Asteraceae, identical inversions have recently been documented in *Leibnitzia* Cass. [42,43]. Given that simple sequence repeats (SSRs) are known to promote plastome instability and structural variation [13,44,45], the presence of mononucleotide SSRs within this inversion region further supports the potential for structural plasticity in Asteraceae plastomes. To evaluate the evolutionary implications of this inversion, we performed selection tests on the affected protein-coding genes. The results showed negative dN/dS ratios, suggesting strong purifying selection despite the structural rearrangement. This indicates that these genes maintain essential functional roles, even under structural modifications. While the coding regions remain highly conserved, genome rearrangements may still provide evolutionary flexibility by disrupting non-coding or intergenic regions, potentially affecting gene expression regulation [46,47]. This structural plasticity could contribute to ecological adaptation, though further experimental validation is needed to test this hypothesis.

We further annotated a pseudogenized *accD* in the LSC region of *A. himalaicum*. This gene encodes the β-subunit of acetyl-CoA carboxylase (ACCase), a key enzyme in fatty acid biosynthesis [48]. In *A. himalaicum*, an inserted repeat sequence near the stop codon led to premature termination, rendering *accD* nonfunctional. Pseudogenization of *accD* has been reported in diverse plant lineages, including *Orobanchaceae* Vent. [49] and *Cactaceae* Juss. [50]. Comparative genomic analyses revealed that the *accD* sequences in these taxa exhibit extensive insertions/deletions (indels) and high nucleotide substitution rates, suggesting its function may have been replaced by homologs in the nuclear genome [51,52]. This phenomenon is particularly common in heterotrophic or parasitic plants with reduced reliance on plastid-based metabolism, which has been named as a functional compensation mechanism in plants [53,54]. Indeed, nuclear genomes can compensate for plastid gene loss through paralogous genes, as observed in *Cuscuta* L. and other taxa [55,56].

Positive selection plays a pivotal role in driving organismal adaptation to diverse and changing environments, while purifying selection acts as a fundamental conservative force maintaining genomic stability and functional integrity [57,58,59]. In our study, all protein-coding genes exhibited signatures of purifying selection across populations, consistent with findings in most reported angiosperm plastomes [60]. This genomic conservation is likely related to the uniparental inheritance of plastids [61], which limits genetic diversity and reduces opportunities for homologous recombination. In addition, plastomes have undergone the substantial evolutionary simplification of plastomes from their ancestral state, primarily retaining housekeeping genes involved in essential functions [62].

### 3.2. Genetic Diversity, Genetic Structure and Conservation Implications

Our pan-plastome analysis indicates that *A. himalaicum* exhibits a lower genetic diversity (Pi = 0.002) compared to other medicinal herbaceous species, such as *Hemerocallis citrina* Baroni, and *Pistacia chinensis* Bunge [23,63]. This reduced variation is likely related to restricted gene flow, as evidenced by significant isolation by distance (IBD, r = 0.4691, *p* = 0.001). Additionally,, the maternally inherited chloroplast DNA reveals limited seed dispersal capacity, leading to pronounced population differentiation [64,65]. In contrast, nuclear patterns may suggest more extensive pollen-mediated gene flow that requires further investigation. Based on the phylogenetic tree and principal component analysis (PCA), the 87 individuals from 18 populations were divided into three lineages (Figure 2), including populations from northeastern China and the Korean Peninsula (Group A), populations from southern China (Group B), and populations from Japan (Group C). The divergence between Group A and Group B occurred at 35–45° N (Figure 2), coinciding with the position of a north–south genetic split of *Juglans* L., *Acer mono* Maxim., and *Lindera obtusiloba* Blume [66,67,68]. Moreover, the separation of Japanese populations (Group C) from continental groups reflects the phylogeographic barrier role of the East Sea, consistent with patterns in *Platycrater arguta* Siebold & Zucc. and *Ligularia hodgsonii* Hook. [60,61,62,63,64,65,66,67,68,69,70,71].

Species distribution modeling for *A. himalaicum* indicated generally stable distributions, although highly suitable areas slightly decreased during the LGM period (Figure 5). According to the predicted region during the LGM, the optimal distribution areas of *A. himalaicum* showed three distinctly isolated areas in southern China and northeastern China, along with the Korean Peninsula and Japanese regions, supporting an intraspecific differentiation event that occurred earlier than the LGM. The southern regions are primarily concentrated around the Sichuan Basin and southeastern Himalayas, which can potentially be explained by the lower ice sheet that developed across the Qinghai-Tibetan Plateau and adjacent mountain ranges [72,73,74,75]. *A. himalaicum* harbors five distinct haplotypes in the Sichuan Basin, indicating considerable genetic variation within the region. Studies have revealed that the surrounding area of the Sichuan Basin in China was a multiple glacial refuge for many plants (e.g., *Primula ovalifolia* Franch. [76], *Dysosma versipellis* (Hance) M. Cheng [77], *Davidia involucrata* Baill. [78], *Populus lasiocarpa* Oliv. [74]), but we failed to find a very high genetic diversity and haplotype distribution in this region. The unexpectedly low nucleotide diversity suggests a complex evolutionary history, such as recent colonization from a small founder population, limiting time for mutation accumulation [79,80]. Moreover, demographic bottlenecks prior to colonization may have eroded ancestral diversity [81]. Accordingly, the suitable distribution areas shared by multiple periods of a species across them are also inferred to be refuges. *A. himalaicum* is accustomed to growing in shady and dense environments; the regions of the surrounding Sichuan Basin have abundant rainfall and frequent rainy seasons that are beneficial for their growth [82,83]. From this perspective, the mountain ranges surrounding the Sichuan Basin have always been the most suitable habitats for *A. himalaicum* all the time, possibly indicating their potential status as refuges [70,84]. In contrast to southern lineage, the populations in Japan exhibit significant loss of high-latitude habitats and severe habitat fragmentations of *A. himalaicum*, reflecting a southward contraction under cooling conditions, as with other species [85,86].

Based on our genetic findings, we propose the recommendations for the conservation and sustainable utilization of *A. himalaicum*. First, in situ conservation strategies should be implemented for the three evolutionary units, with priority given to unique genetic compositions and marginal populations in the Korean Peninsula and southern populations. Second, germplasm banks encompassing all three lineages should be established, with separate cultivation maintained for each phylogroup to preserve their genetic integrity. Regarding sustainable utilization, we recommend prioritizing the screening of medicinal bioactive compounds in southern populations and Japanese populations while considering controlled cross-lineage breeding to enhance genetic diversity.

## 4. Materials and Methods

### 4.1. Plant Materials and Plastome Assembly

Based on the herbarium records, field investigations, and literature of *Adenocaulon himalaicum* Edgew., a total of 87 representative individuals from 18 populations were collected from China, Japan, and Korea, covering its known distribution range (Figure 1). For each population, fresh leaves were sampled from 3–6 individuals and stored in silica gel within each population. In addition, one individual of *A. nepalense* Bittmann. from Nepal was collected as an outgroup. Voucher specimens for populations were labeled, catalogued, and stored in the Herbarium of Henan Agricultural University. Genomic DNA was extracted using the CTAB method [87], and quality assessment for purity, concentration, and integrity used the NanoPhotometer^®^ spectrophotometer (Implen, WV, USA) and 1% agarose gel electrophoresis. Qualified DNA was then fragmented for paired-end library construction following a standard protocol in the Biomarker Technologies (Beijing, China). High-throughput sequencing was subsequently performed on the DNBSEQ-T7 platform (MGI, Beijing, China). To extract plastome reads, the raw reads were mapped to the reference plastome of the close species, *Famatinanthus decussatus* (Hieron.) Ariza & S.E.Freire (NC_081938) and (*Leibnitzia anandria* (L.) Turcz. (PP566209) using Bowtie2 v2.5.4 [88] and SAMtools v1.15 [89]. The obtained paired reads for each individual were assembled using NOVOPlasty v4.3.1 [90] with the kmer setting as 31. The assembled plastomes were initially annotated using the CPGAVAS2 [91] platform. Then all the plastomes were manually checked for start and stop codons in Geneious Prime v2025 with the above two closely related species used as a reference. Finally, OGDRAW v1.3.1 [92] was used to draw the physical map of the plastomes with a default parameter.

### 4.2. Comparative Analysis of the Pan-Plastome

The pan-plastomes of *A. himalaicum* were aligned using MAFFT v7.526 with a default parameter [93], and the genome size, gene content, and quadripartite structure were compared and summarized using Geneious Prime v2025. The single nucleotide variables (SNVs) and indels were determined to infer the genomic variants across *A. himalaicum* using DnaSP v6 and then were manually checked [94]. The relative synonymous codon usage (RSCU) of protein-coding genes in each plastome was calculated using CodonW v1.4.2 [95] to assess codon usage bias with a default parameter. Tandem repeats were identified using the Tandem Repeats Finder (TRF) v.4.10 [96] with default parameters. Simple sequence repeats (SSRs) were identified using MISA v2.1 [97], with the minimum number of repeat units set to 10 for mononucleotide, 6 for dinucleotide, and 5 for tri-, tetra-, penta-, and hexanucleotide motifs, while repeat types (forward, palindromic, reverse, and complementary) were detected using the REPuter v2.74 [98] online tool under default settings.

To examine genes under selective pressure, non-synonymous substitution rates (dN), synonymous substitution rates (dS), and their ratios (dN/dS) were calculated for protein-coding genes using the CODEML module in PAML v4.9 [99]. The phylogenetic tree of each protein coding gene was constructed by IQ-TREE v2.2 [100] under the GTR model, and codon alignment was performed using MAFFT v7.526 with a default parameter.

### 4.3. Population Structure and Phylogroup Analysis

Given the high conservatism of the coding sequences, whole plastomes with a single copy of the inverted repeating (IR) region were aligned using MAFFT v7.526 with a default parameter [93]. The obtained sequences were used to construct the phylogenetic tree by IQ-TREE v2.2 and MrBayes v3.2.7 [100,101], with *A. nepalense* included for the outgroup. For maximum likelihood (ML) phylogeny in IQ-TREE, the best-fit nucleotide substitution model (TVM + F + I) was selected automatically by ModelFinder [102], and branch support was assessed through 1000 bootstrap replicates and BNNI optimization. For Bayesian inference (BI) phylogeny, the Markov chain Monte Carlo (MCMC) analysis was run for 10 million generations under the best-fit substitution model (GTR + I) selected using PAUP v4.0 and MrModeltest v2.4 [103,104], with trees sampled every 1000 generations. The first 25% of sampled trees were discarded as burn-in, and chain convergence was assessed using TRACER v1.7.1 [105]. In addition, principal component analysis (PCA) was performed for all individuals using TASSEL v5.0 [106].

Haplotype distribution was conducted in DnaSP v6 [94] to detect the number and types of haplotypes within each *A. himalaicum* populations. The obtained haplotypes were imported into PopART v1.7 [107] and a haplotype network was constructed using TCS v1.2 [108] to visualize phylogroups. The phylogenetic relationships among haplotypes were reconstructed using the BI and ML methods implemented in MrBayes v3.2.7 [101] and IQ-TREE v2.2, respectively, with *A. nepalense* as the outgroup. For BI tree, the Markov chain Monte Carlo (MCMC) analysis was run for 10 million generations under the best-fit substitution model (GTR + I), with trees sampled every 1000 generations. The first 25% of sampled trees were discarded as burn-in, and chain convergence was assessed using TRACER v1.7.1 [105]. For ML tree, the best-fit nucleotide substitution model (TVM + F + I) was selected automatically by ModelFinder [102], and branch support was assessed through 1000 bootstrap replicates and BNNI optimization.

### 4.4. Genetic Diversity and Genetic Differentiation

To assess genetic variation within and among populations, the haplotype diversity (Hd) and nucleotide diversity (Pi) were calculated using DnaSP v6 [94] based on the genetic group plastomes of 87 *A. himalaicum* individuals. Total *G*_ST_ and *N*_ST_ were analyzed using PermutCpSSR v2.0 with 1000 permutations to estimate differentiation between populations and test the presence of a phylogeographical structure [109]. Analysis of molecular variance (AMOVA) was conducted using Arlequin v3.5.2 [110] to estimate genetic differentiation indices, including among-group (*F*_CT_), among-population within groups (*F*_SC_), and within-population (*F*_ST_) components, and to quantify the genetic variance partitioned at different hierarchical levels. Pairwise genetic differentiation (*F*_ST_) values between populations were also calculated using Arlequin v3.5.2 [110]. To assess the impact of isolation by distance (IBD) and isolation by environment (IBE) on genetic differentiation, we generated the correlations between the *F*_ST_ matrix and both geographic and environmental matrices, respectively. The geographic distance matrix was estimated based on GPS coordinates using the ‘geosphere v1.5.20’ package in R [111]. For environmental distance, a total of 19 bioclimatic variables were obtained from the WorldClim v2.1 [112] database. Based on the contribution of each factor to species distribution, six environmental variables with correlation coefficients below 0.75 were retained for modeling (Table S9, Figure S6). These variables included annual mean temperature (bio1), mean diurnal range (bio2), isothermality (bio3), temperature seasonality (bio4), mean temperature of warmest quarter (bio10), and annual precipitation (bio12). Environmental distances were calculated using Euclidean distance by points according to the first two principal components (Clim_PC1 and Clim_PC2) of the environmental variables. Mantel tests were performed based on the correlations between genetic distance and both geographic and environmental distance matrices with 999 permutations using the ‘vegan v2.6.10’ package in R [113].

### 4.5. Species Distribution Modeling

To reconstruct the potential distribution range of *A. himalaicum* across different climate scenarios, ecological niche modeling was performed using MaxEnt v3.4.3 [114]. A total of six non-redundant bioclimatic variables, as given above, were obtained from the WorldClim v1.4 [115] database for species distribution modeling. A total of 194 occurrence records from herbarium databases, field surveys, and published literature were collected and checked for the following analysis, with 10 replicates under 75% training data and 25% testing data. Although the more recent WorldClim v2.1 [112] provides contemporary (1970–2000 baseline) and future climate data based on multiple GCMs, paleoclimatic layers are not available. Therefore, we used WorldClim v1.4 [115] for historical periods, including climate layers of the Last Glacial Maximum (LGM, ca. 22,000 years ago) and Mid-Holocene (ca. 6000 years ago), under two climate models (MIROC-ESM and CCSM4) at a resolution of 2.5 arc-minutes to ensure comparability across different periods.

## 5. Conclusions

This study demonstrates the effectiveness of the pan-plastome approach in resolving intraspecific divergence and population genetic structure in *Adenocaulon himalaicum*, uncovering three distinct genetic lineages shaped by geographic isolation. The pronounced genetic structure highlights the necessity for lineage-specific conservation strategies, with particular attention to marginal populations that may harbor unique genetic adaptations and enhanced medicinal properties. By assembling the first comprehensive pan-plastome for *A. himalaicum*, this study provides critical insights into plastome evolution and population-level diversification while establishing a genomic framework for future breeding schemes and sustainable use of this medicinal species. Integrating nuclear genome analyses with landscape genomic approaches would enable robust reconstruction of historical demography, identification of gene flows, and comprehensive characterization of adaptive genomic architecture, which provide a critical foundation for evidence-based conservation planning and targeted domestication strategies.

## Data Availability

The raw datasets used during the current study have been submitted to the National Center for Biotechnology Information under project NO. PRJNA1285360. The chloroplast genome data assembled in this study have been submitted to the National Center for Biotechnology Information under NO. PX241414-PX241501.

## Supplementary Materials

The following supporting information can be downloaded at: https://www.mdpi.com/article/10.3390/ijms26178594/s1.
