# Supplementary material for: Pan-Plastome Analysis Reveals the Genetic Diversity and Genetic Divergence of Adenocaulon himalaicum (Asteraceae)
Source: Int J Mol Sci. 2025 Sep 4;26(17):8594. doi: 10.3390/ijms26178594 (PMC12429270; doi:10.3390/ijms26178594)

Table S1 The detailed formation for population characteristics and genetic diversity parameter of 18 *A. himalaicum* populations analyzed in this study, including population code, number of individuals, geographic coordinates (latitude/longitude), sequencing coverage, nucleotide diversity (Pi), haplotype diversity (Hd), and haplotype distribution.

**Population code**	**Number of individuals**	**Latitude/ Longitude**	**Sequencing coverage**	**Pi*10³**	**Hd**	**Haplotypes**
AJH	5(ade1-ade5)	44.04N,127.75E	486.9 - 1653.5	0.000	0.000	Hap18(5)
AK	5(ade6-ade10)	33.69N,107.86E	197.1 – 343.4	0.000	0.000	Hap1(5)
AT	5(ade11-ade15)	42.40N,128.09E	228.8 - 934	0.030	0.600	Hap18(3), Hap4(2)
DLJ	3(ade16-ade18)	27.70N,98.34E	549.2 – 739.6	0.000	0.000	Hap17(3)
GS	5(ade19-ade23)	34.37N,106.00E	108.4 - 346	0.000	0.000	Hap16(5)
HZ	5(ade24-ade28)	32.74N,106.87E	374.8 – 953.5	0.000	0.000	Hap1(5)
JFS	5(ade29-ade33)	29.03N,107.18E	262.6 – 624.6	0.000	0.000	Hap15(5)
KOO	5(ade34-ade38)	35.90N,127.34E	141.8 – 604.9	0.000	0.000	Hap4(5)
LC	5(ade39-ade43)	33.72N,111.65E	162.1 – 564.6	0.000	0.000	Hap4(5)
PQG	5(ade49-ade53)	37.84N,111.45E	181.4 – 746.9	0.003	0.400	Hap13(4), Hap14(1)
QY	5(ade54-ade58)	41.84N,124.94E	336.3 – 1173.8	0.030	0.400	Hap11(4), Hap12(1)
S26	3(ade95-ade97)	34.82N,133.12E	3281.2 – 4014.2	0.000	0.000	Hap2(3)
SLJ	5(ade59-ade63)	31.42N,110.33E	14.5 – 330.3	0.060	0.400	Hap9(1), Hap10(4)
SZ	5(ade64-ade68)	42.40N,127.65E	156.7 – 305.4	0.030	0.400	Hap5(4), Hap8(1)
TB	6(ade69-ade73, ade98)	33.99N,107.67E	170.5 - 877	0.020	0.333	Hap1(5), Hap7(1)
XZ	5(ade74-ade78)	30.02N,94.63E	232.9 - 868	0.000	0.000	Hap6(5)
YB	5(ade79-ade83)	42.47N,128.66E	413 – 2184.8	0.030	0.400	Hap4(4), Hap5(1)
YL	5(ade84-ade88)	25.66N,99.13E	467.3 – 2724.7	0.000	0.000	Hap3(5)

Table S2 Characteristics of quadripartite structure (i.e., length of large single-copy (LSC), small single-copy (SSC) and inverted repeat (IR)) of the pan-plastome in 87 *A. himalaicum* individual.

Sample	Population	LSC_Length (bp)	IR_Length (bp)	SSC_Length (bp)	total_Length (bp)
ade01	AJH	83334	25112	18642	152200
ade02	AJH	83333	25112	18642	152199
ade03	AJH	83334	25112	18642	152200
ade04	AJH	83333	25112	18642	152199
ade05	AJH	83333	25112	18642	152199
ade06	AK	83311	25118	18641	152188
ade07	AK	83311	25118	18641	152188
ade08	AK	83311	25118	18641	152188
ade09	AK	83311	25118	18641	152188
ade10	AK	83312	25118	18641	152189
ade11	AT	83333	25112	18642	152199
ade12	AT	83333	25112	18642	152199
ade13	AT	83333	25112	18642	152199
ade14	AT	83332	25112	18637	152193
ade15	AT	83332	25112	18637	152193
ade16	DLJ	83314	25112	18643	152181
ade17	DLJ	83314	25112	18643	152181
ade18	DLJ	83314	25112	18643	152181
ade19	GS	83332	25112	18642	152198
ade20	GS	83332	25112	18642	152198
ade21	GS	83331	25112	18642	152197
ade22	GS	83332	25112	18642	152198
ade23	GS	83332	25112	18642	152198
ade24	HZ	83312	25118	18641	152189
ade25	HZ	83311	25118	18641	152188

ade26	HZ	83311	25118	18641	152188
ade27	HZ	83311	25118	18641	152188
ade28	HZ	83311	25118	18641	152188
ade29	JFS	83306	25118	18641	152183
ade30	JFS	83306	25118	18641	152183
ade31	JFS	83306	25118	18641	152183
ade32	JFS	83306	25118	18641	152183
ade33	JFS	83306	25118	18641	152183
ade34	KOO	83332	25112	18642	152198
ade35	KOO	83332	25112	18642	152198
ade36	KOO	83332	25112	18642	152198
ade37	KOO	83332	25112	18642	152198
ade38	KOO	83330	25112	18642	152196
ade39	LC	83333	25112	18642	152199
ade40	LC	83333	25112	18642	152199
ade41	LC	83333	25112	18642	152199
ade42	LC	83333	25112	18642	152199
ade43	LC	83333	25112	18642	152199
ade49	PQG	83332	25112	18642	152198
ade50	PQG	83332	25112	18642	152198
ade51	PQG	83332	25112	18642	152198
ade52	PQG	83332	25112	18642	152198
ade53	PQG	83332	25112	18642	152198
ade54	QY	83332	25112	18642	152198
ade55	QY	83332	25112	18642	152198
ade56	QY	83332	25112	18637	152193
ade57	QY	83332	25112	18642	152198
ade58	QY	83332	25112	18642	152198
ade59	SLJ	83313	25118	18641	152190
ade60	SLJ	83313	25118	18641	152190
ade61	SLJ	83313	25118	18641	152190

ade62	SLJ	83313	25118	18641	152190
ade63	SLJ	83312	25118	18641	152189
ade64	SZ	83332	25112	18642	152198
ade65	SZ	83332	25112	18642	152198
ade66	SZ	83332	25112	18642	152198
ade67	SZ	83332	25112	18642	152198
ade68	SZ	83332	25112	18642	152198
ade69	TB	83311	25118	18641	152188
ade70	TB	83312	25118	18641	152189
ade71	TB	83310	25118	18641	152187
ade72	TB	83311	25118	18641	152188
ade73	TB	83252	25118	18641	152129
ade74	XZ	83295	25119	18638	152171
ade75	XZ	83295	25119	18638	152171
ade76	XZ	83295	25119	18638	152171
ade77	XZ	83295	25119	18638	152171
ade78	XZ	83295	25119	18638	152171
ade79	YB	83329	25112	18642	152195
ade80	YB	83331	25112	18642	152197
ade81	YB	83331	25112	18642	152197
ade82	YB	83332	25112	18642	152198
ade83	YB	83329	25112	18642	152195
ade84	YL	83294	25119	18643	152175
ade85	YL	83294	25119	18643	152175
ade86	YL	83294	25119	18643	152175
ade87	YL	83294	25119	18643	152175
ade88	YL	83295	25119	18643	152176
ade95	S26	83341	25112	18642	152207
ade96	S26	83336	25112	18642	152202
ade97	S26	83336	25112	18642	152202
ade98	TB	83311	25118	18641	152188

Table S3 All the 113 genes annotated from *A. himalaicum* plastomes, including protein-coding genes, tRNA genes and rRNA genes. Genes with two introns were marked with asterisk.

Gene Category	Functional Group	Gene Name
Protein-coding Genes	Photosystem I	*psaA, psaB, psaC, psaI, psaJ*
	Photosystem II	*psbA, psbB, psbC, psbD, psbE, psbF, psbH, psbI, psbJ, psbK, psbT, psbL, psbZ, psbM, psbN*
	Small subunit of ribosome	*rps2, rps3, rps4, rps7, rps8, rps11, rps12*, rps14, rps15, rps16, rps18, rps19*
	Large subunit of ribosome	*rpl2, rpl14, rpl16, rpl20, rpl22, rpl23, rpl32, rpl33, rpl36*
	NADH dehydrogenase	*ndhA, ndhB, ndhC, ndhD, ndhE, ndhF, ndhG, ndhH, ndhI, ndhJ, ndhK*
	Cytochrome b/f complex	*PetA, petB, petD, petG, petL, petN*
	ATP synthase	*atpA, atpB, atpE, atpF, atpH, atpI*
	RNA polymerase	*rpoA, rpoB, rpoC1, rpoC2*
	Large subunit of Rubisco	*rbcL*
	Unknown function	*ycf1, ycf2, ycf3*, ycf4*
	Cytochrome c biogenesis protein	*ccsA*
	Envelope membrane protein	*cemA*
	Subunit of ATP-dependent Clp	*clpP**
	Translation initiation factor	*infA*
	Subunit of acetyl-CoA	*AccD*
	Maturase	*matK*
tRNA genes	Transfer RNA	*trnA-UGC, trnC-GCA, trnD-GUC, trnE-UUC, trnF-GAA, trnG-UCC, trnG-GCC, trnH-GUC, trnI-CAU, trnI-GAU, trnK-UUU, trnL-CAA, trnL-UAA, trnL-UAG, trnM-CAU, trnN-GUU, trnP-UGG, trnQ-UUG, trnR-ACG, trnR-UCU, trnS-GGA, trnS-GCU, trnS-UGA, trnT-GGU, trnT-UGU, trnV-GAC, trnV-UAC, trnW-CCA, trnY-GUA, trnfM-CAU*
rRNA genes	Ribosomal RNA	*rrna 4.5, rrna 5, rrna 16, rrna 23*

Table S4 Statistics on the location of simple sequence repeats (SSRs) in *A. himalaicum* plastomes, including protein--coding genes and intergenic regions.

regions	Count
ycf1	348
rpoB	87
rpoC1	87
clpP1	87
rpoA	87
atpH - atpF	87
ndhC - trnV-UAC	87
psaA - ycf3	87
psbZ - trnG-GCC	87
rpl32 - trnL-UAG	87
rpl33 - rps18	87
rps19 - ycf1	87
rps8 - rpl14	87
trnG-GCC - trnfM-CAU	87
trnT-UGU - trnL-UAA	87
trnH-GUG - psbA	86
atpF - atpA	82
atpB - rbcL	81
psaI - ycf4	81
psbC - trnS-GGA	79
rpoC2 - rps2	74

rpl20 - rps12	64
rpoA - rps11	26
ccsA - ndhD	5
petA - psbJ	3
trnF-GAA - ndhJ	3

Table S5 Distribution of SNVs in the plastomes of *A. himalaicum* across plastome regions (LSC, SSC, IR) and functional regions (protein-coding regions, introns, and intergenic regions).

regions	SNVs	percent
LCS	78	67.2%
SSC	28	24.1%
IR	10	8.6%
protein--coding region	71	61.2%
intron	0	0
intergenic regions	45	38.8%

Table S6. Statistics of genetic diversity and neutrality test results for all populations of *A. himalaicum*, with values for haplotype number, haplotype diversity, nucleotide diversity (Pi), parsimony-informative sites, and N_{ST}/G_{ST} (p-values)

Parameters	Value
Number of haplotypes	18
Haplotype diversity	0.913
Pi	0.0002
Parsimony informative sites	116
N_{ST} / G_{ST} (pvalue)	0.880/0.773 ($p<0.05$)

Table S7 Analysis of molecular variance (AMOVA) based on the plastome sequences for *A. himalaicum*.

Source of variation	Sum of squares	Variance components	Percentage of variation	Fixation indices
Among groups	2156.295	44.21125	73.43	F_{CT} = 0.87032 (p<0.01)
Within groups	1061.296	13.92135	23.12	F_{SC} = 0.96555 (p<0.01)
Within populations	143.133	2.07440	3.45	F_{ST} = 0.73432 (p<0.01)

Table S8 AUC values for species distribution modelling of *A. himalaicum* of the Last Glacial Maximum (LGM, ca. 22,000 years ago), Mid-Holocene (ca. 6000 years ago) under two climate models (MIROC-ESM and CCSM4) scenario, and under present climate conditions (1970–2000).

Area (×10^4 km^2)	AUC
current	0.914 (p=0.006)
LGM-MR	0.918 (p=0.006)
LGM-CC	0.923 (p=0.005)
Mid Holocene-MR	0.919 (p=0.003)
Mid Holocene-CC	0.917 (p=0.008)

Table S9 The contribution of 19 climatic variables to the distribution of *A. himalaicum* and explanations of each climatic variable.

Variable	Environmental variable	Percent contribution (%)
Bio12	Annual Precipitation (mm)	34.9
Bio10	Mean Temperature of Warmest Quarter (℃)	19.8
Bio1	Annual Mean Temperature (℃)	8.4
Bio3	Isothermality (Bio2/Bio7) (×100)	7.5
Bio11	Mean Temperature of Coldest Quarter (℃)	6.3
Bio14	Precipitation of Driest Month (mm)	5.2
Bio8	Mean Temperature of Wettest Quarter (℃)	4
Bio9	Mean Temperature of Driest Quarter (℃)	2.4
Bio4	Temperature Seasonality (standard deviation ×100)	2.4
Bio2	Mean Diurnal Range (Mean of monthly (max temp - min temp)) (℃)	2.1

Bio7	Temperature Annual Range (Bio5-Bio6) (℃)	2.1
Bio13	Precipitation of Wettest Month (mm)	1.2
Bio6	Min Temperature of Coldest Month (℃)	1
Bio17	Precipitation of Driest Quarter (mm)	0.9
Bio18	Precipitation of Warmest Quarter (mm)	0.6
Bio15	Precipitation Seasonality (Coefficient of Variation)	0.5
Bio16	Precipitation of Wettest Quarter (mm)	0.4
Bio19	Precipitation of Coldest Quarter (mm)	0.3
Bio5	Max Temperature of Warmest Month (℃)	0

Figure S1 Variation in ratio of non-synonymous substitution rate (dN)/synonymous substitution rate (dS), for 79 protein-coding genes in the plastome of *A. himalaicumm.*

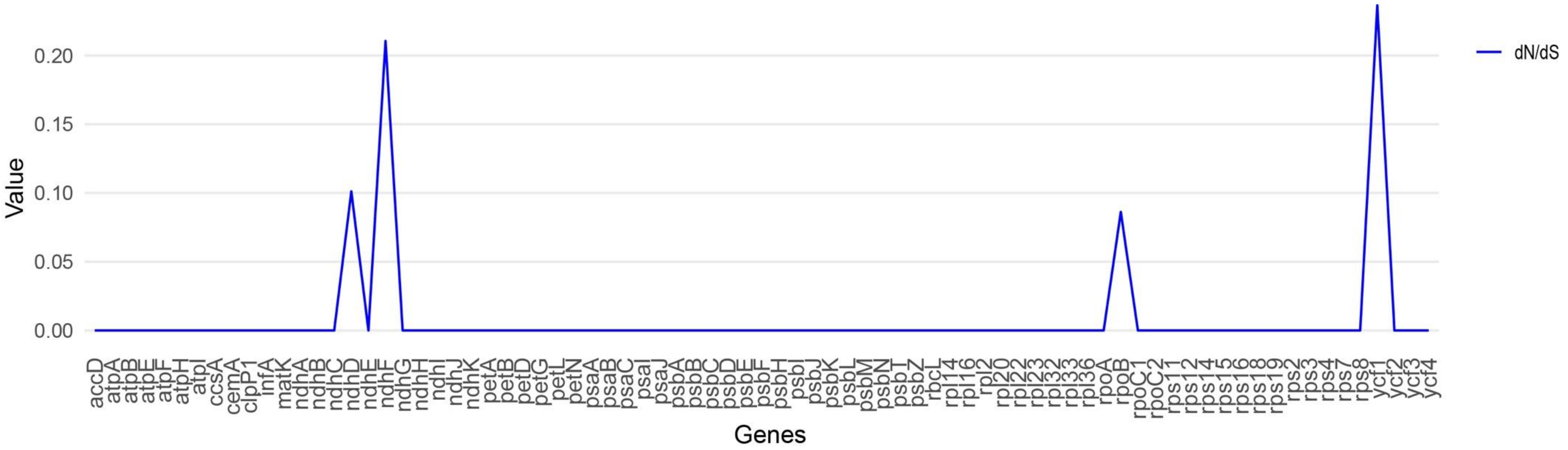

Figure S2 Heat map of relative synonymous codon usage (RSCU) values of protein-coding genes in the plastome of *A. himalaicumm.* The vertical axis represents different individuals, while the horizontal axis denotes codons and their corresponding amino acids. Higher red intensity denotes larger RSCU values, whereas higher blue intensity denotes smaller RSCU values. The Codons with RSCU > 1 are marked with an asterisk.

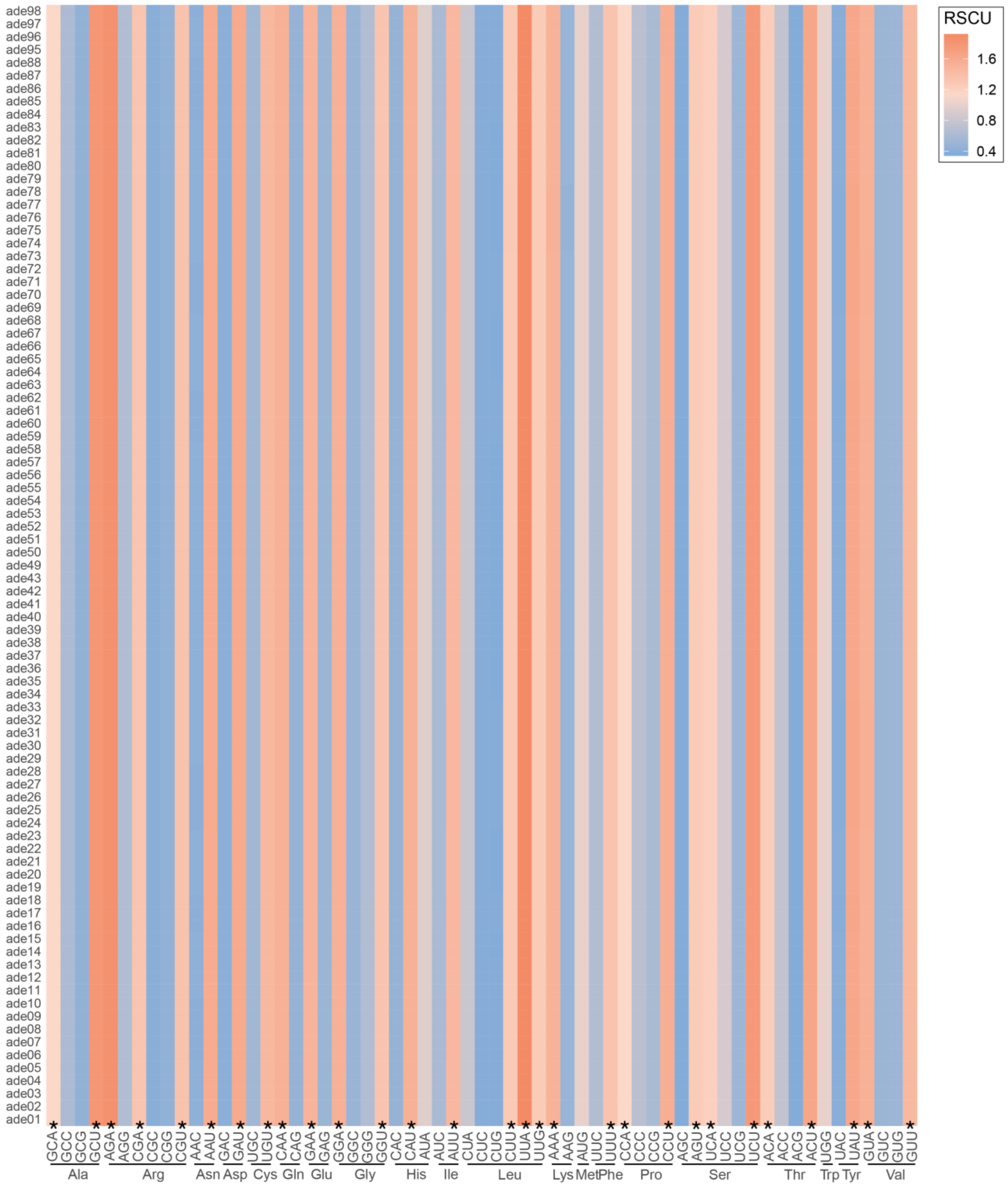
RSCU
1.6
1.2
0.8
0.4
ade98
ade97
ade96
ade95
ade88
ade87
ade86
ade85
ade84
ade83
ade82
ade81
ade80
ade79
ade78
ade77
ade76
ade75
ade74
ade73
ade72
ade71
ade70
ade69
ade68
ade67
ade66
ade65
ade64
ade63
ade62
ade61
ade60
ade59
ade58
ade57
ade56
ade55
ade54
ade53
ade52
ade51
ade50
ade49
ade43
ade42
ade41
ade40
ade39
ade38
ade37
ade36
ade35
ade34
ade33
ade32
ade31
ade30
ade29
ade28
ade27
ade26
ade25
ade24
ade23
ade22
ade21
ade20
ade19
ade18
ade17
ade16
ade15
ade14
ade13
ade12
ade11
ade10
ade09
ade08
ade07
ade06
ade05
ade04
ade03
ade02
ade01
GCA GCC GCG GCU
Ala
AGA AGG CGA CGC CGG CGU
Arg
AAC AAU
Asn
GAC GAU
Asp
UGC UGU
Cys
CAA CAG
Gln
GAA GAG
Glu
GGA GGC GGG GGU
Gly
CAC CAU
His
AUA AUC AUU
Ile
CUA CUC CUG CUU UUA UUG
Leu
AAA AAG
Lys
AUG
Met
UUC UUU
Phe
CCA CCC CCG CCU
Pro
AGC AGU UCA UCC UCG UCU
Ser
ACA ACC ACG ACU
Thr
UGG
Trp
UAC UAU
Tyr
GUA GUC GUG GUU
Val

Figure S3 Summarization of repeat sequences annotated in *A. himalaicum*. (A) Frequency of different SSR motif types; (B) Distribution of repeat types identified by REPuter, including forward (F), palindromic (P), reverse (R), and complementary (C) repeats; (C) long tandem repeat sequences.

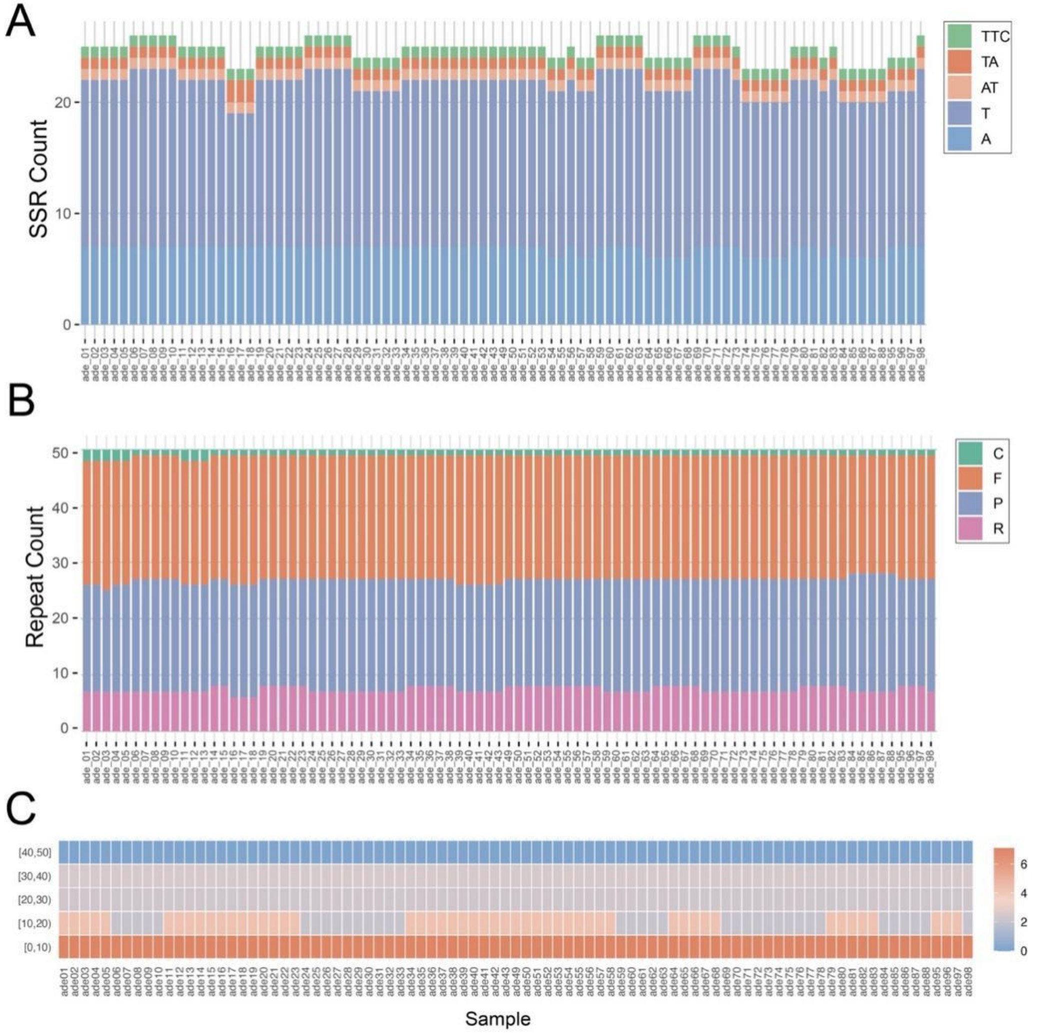

Figure S4 Bayesian phylogenetic tree of *A. himalaicum* populations, with *A. nepalense* as the outgroup. Posterior probabilities (PP) are indicated by circle color.

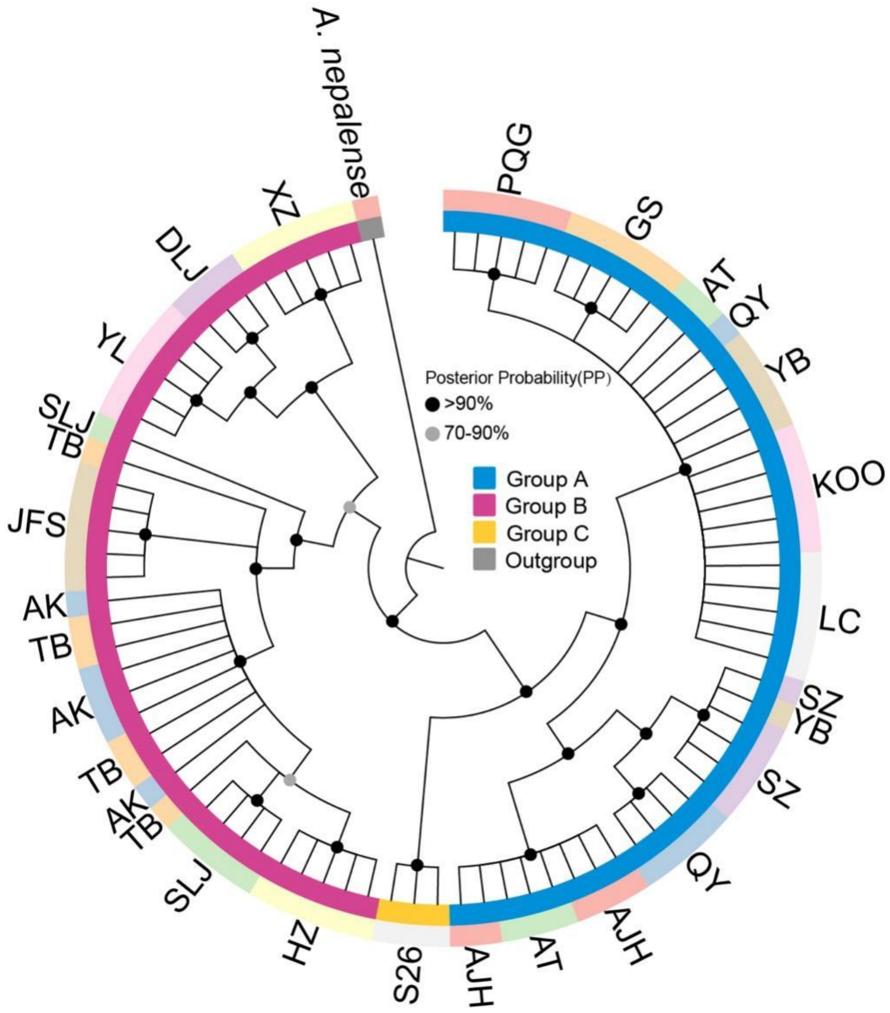

Figure S5 Maximum likelihood (ML) phylogenetic tree of 18 cpDNA haplotypes in *A. himalaicum,* with *A. nepalense* (Hap19) as outgroup. Bootstrap values are shown at the nodes.

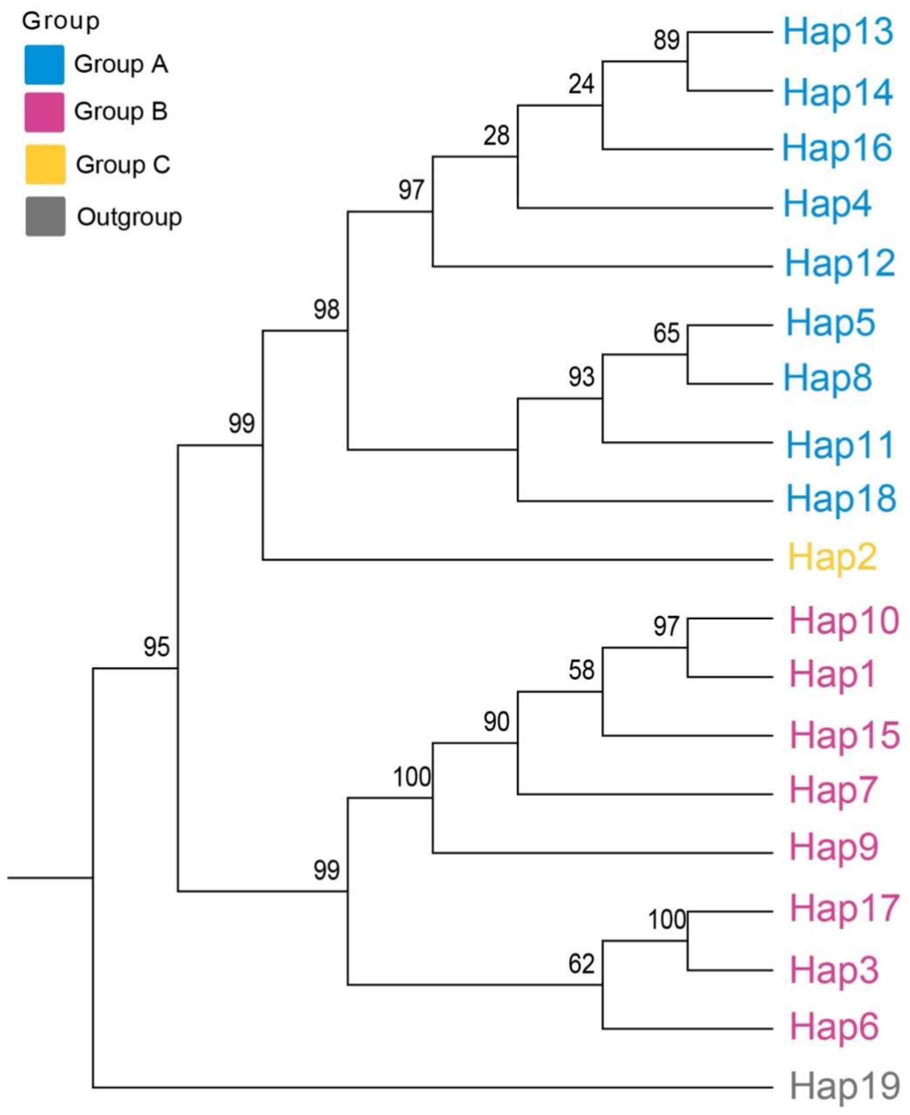
Group
Group A
Group B
Group C
Outgroup
Hap13
Hap14
Hap16
Hap4
Hap12
Hap5
Hap8
Hap11
Hap18
Hap2
Hap10
Hap1
Hap15
Hap7
Hap9
Hap17
Hap3
Hap6
Hap19
89
24
28
97
98
65
93
99
95
97
58
90
100
99
100
62

Figure S6 Correlation heatmap of climatic factors used in *A. himalaicum* distribution modeling. The circles in the upper triangular cells depict pairwise correlations among climatic variables. Circle size is proportional to the absolute value of the correlation coefficient. Circles with red are denoting positive correlations while the blue circles are denoting negative correlations.

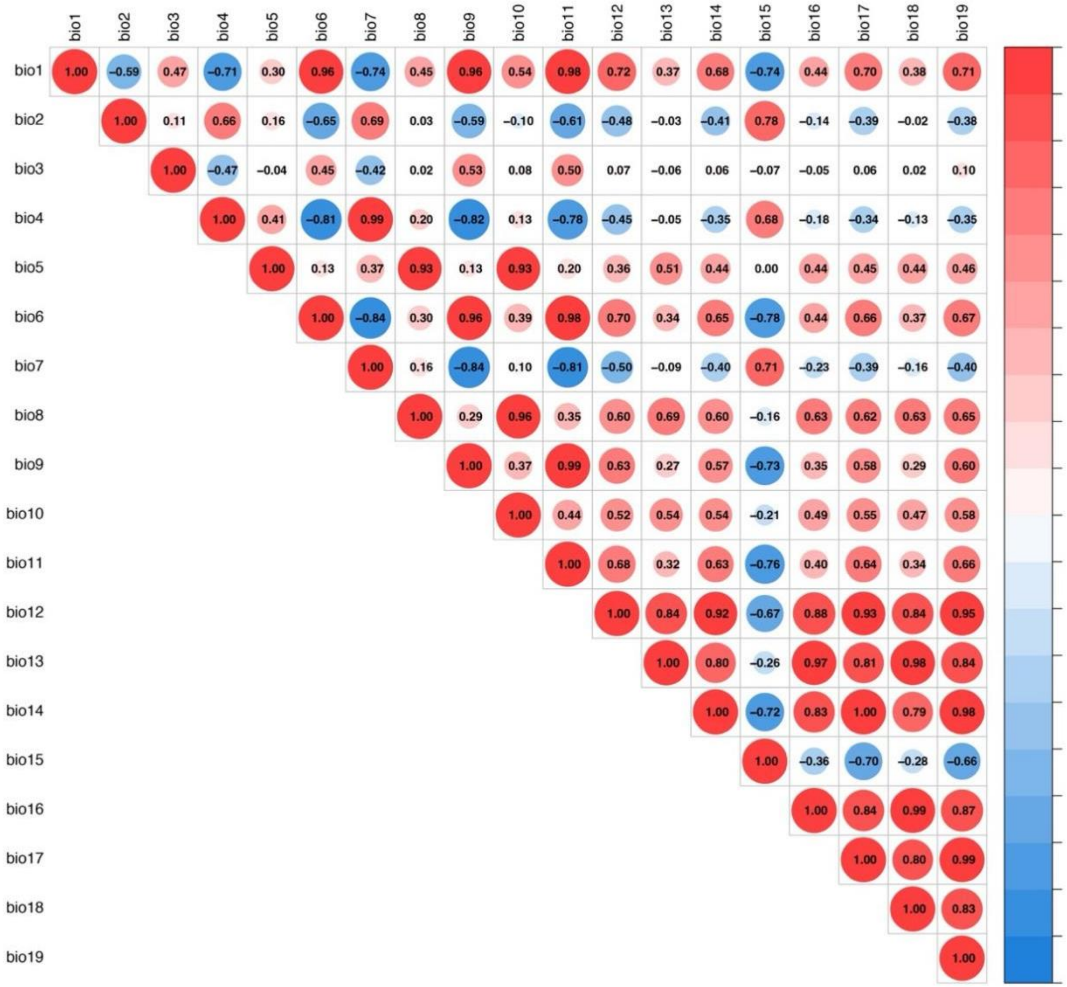

Supplement: Supplementary file 1 [file ijms-26-08594-s001.zip › ijms-3759263-supplementary.pdf]
